# Obstetriciana

**Published:** 1846-06

**Authors:** B. Dowler


9. *Obstetriciana.*—The title of this paper I use as a sort of *carte blanche*, to ramble where I please, without attempting to write a monograph. The artist who painted a sign with a horse on one side and a bear on the other, finding that the people could not tell the difference, wrote the name under each. This I shall not do with the *dramatis personæ* in these sketches, most of whom are beyond the reach of praise or blame. The "all-atoning power of the grave" generally silences malice itself. But to correct errors affecting health and life, to turn away occasionally from the realms of romance and its imaginary woes, to the realities of life; in a word, to consider the silent but courageous sufferings of woman, with the aim of lessening evils not wholly removable, are objects of greater importance than the triumphs of Alexander, or the discoveries of Newton.

One of the most unpleasant features in country practice, originates in the great importunity among patients and their friends for delivering with celerity, requiring almost constant efforts, or seeming efforts for this end, otherwise the accoucheur may lose the confidence of all present, perhaps encounter their displeasure. In towns, thanks to the superior intelligence of the ladies, this is seldom the case. The doctor is not perpetually required in the bed-room, unless in certain emergencies. Where nothing is required he does nothing. This importunity leads to the untimely administration of ergot, which causes the death of many children. Many midwives, as ignorant of anatomy as the simple stargazer is of astronomy, desirous of expediting the labor, seize any salient point of the child, or of the organs of generation of the mother—they pull! On the 19th of January, 1832, I was called to see Mrs. M., who had been delivered three days previously by a midwife, who had pulled the external organs so as to cause mortification, from which she recovered with difficulty. Much of the vagina sloughed. Another midwife, in a breech case, pulled by the genitals of a male child, until they were torn off, causing its death in a few days after birth. On the 27th of Nov., 1832, I was called to see Mrs. R., 15 miles distant in the country, whom I had attended with success in a former labor. Mr. R., like some other farmers, kept a set of tools for making shoes in rainy weather. No sooner had he started for a doctor, than the midwife, in whose care he had left his lady, went to the shoemaker's bench and selected a large pair of pincers, with which she seized the skin of the child's head, which had presented in the usual manner; she tore open the vertex, and exposed the brain. When I arrived, after some hesitation, she brought the child from one corner of the room, where it had been covered up, but did not offer any explanation. The child lingered two days in great misery. I doubt whether this woman suffered any loss of practice by this murder. Indeed, the frequent vivisection of children, during childbirth, under pretences of various kinds, is very apt to enhance the reputation of the male practitioner. I could name ladies whose children have been dissected under the pretence that the pelvic bones are deformed, or too narrow, and who since have had large living children, and some of whom I have delivered with the greatest facility myself. For the honor of our profession, it is hoped that such cases are not often met with, though MM. Bayle and Gibert declare, that in France, especially in large cities, the most popular accoucheurs are those possessing the least merit and probity. (*Dict. de Méd.*, *Art. Accoucheur.*)

In obstetrics, ignorance causes more mischief than dishonesty. A very amiable young woman, an only child, during her first labor, had a convulsion, while an old accoucheur of much reputation was attempting to deliver her. In order to prevent her from biting her tongue, her father held her teeth asunder, and, during her struggles, dislocated her jaw. After being attended by two aged accoucheurs and physicians, who gave her sundry medicines without dreaming of a luxation, I was called 37 days after the accident, and reduced her jaw at once. The displaced bones had formed bad ulcers within the cheeks; purges and slops (for of course she could not chew anything during 37 days) had reduced her strength and blooming appearance.

A midwife, very old and celebrated in her vicinity—more than a quarter of a century in practice—seven miles from town (Clarksburg, Virginia), requested my services for "a falling out of the womb," as she called it, in her own person, of thirty years' standing, which, as she related, often became dry, tender, swelled, and so painful as sometimes to cause fainting, and had recently kept her wholly in bed. I found a pudendal hernia, which had descended within the pelvis, opposite the acetabulum, distending the right labium, pushing the vagina to the opposite side, displacing the womb in that direction. The womb was high up, and in all respects natural—a portion of the vagina had descended, covering the intestine. A midwife, ignorant of the position, size, and properties of the womb! her own womb!

Another midwife of much practice (peace to her *manes!* I owe her no ill-will—with her dying breath, she bequeathed to me the obstetrical jurisdiction over her children and grandchildren), made it a rule to keep the patient awake the whole of the first night after an accouchement. She placed watchers at the bed-side to prevent sleep, in order to insure good luck to the mother and child!—a practice as cruel as dangerous, after the tumultuous actions, moral, mental, and physical, incidental to labor—actions followed by exhaustion, for which sleep is the best remedy—actions which lay the foundation of febrile movements, when the rest is disturbed. In such cases Sancho Panza's benediction "on the inventor of sleep" is well merited, as all ought to know. Dr. Rigby, in his obstetrical lectures, says, that in Germany there is an old saying, that a *primipara* [she that is delivered of her first child] ought not to be allowed to sleep for the first twelve hours after her labor. These Germans have some odd notions in New Orleans. In several instances at my first visit after delivery, I have found the children bandaged like mummies from the feet up, including the arms, which are placed against the sides. The whole chest is so tight, that the child sucks with difficulty in some cases. The body is almost rigid from the great amount and tightness of the bandages. I have never been able to induce the parents to remove them, until after a certain number of days. The theory is a very simple one—the child was crooked before birth, and requires straightening while its bones are soft. It has so happened that these children have done very well; they proved remarkably straight, active, and healthful, in a few cases which I have observed. Perhaps this was accidental.

Not long since, a German midwife, who had delivered a watchmaker's lady ten days before of a child, letting the placenta remain all that period, requested my aid—to bear the responsibility, I ought to say, of the woman's death. She had flooded from day to day; the arteries had ceased to beat in the extremities; she was nearly speechless—could scarcely swallow a little cordial; all the tissues, even the tongue and gums, were cold and blanched. A large placenta, nearly putrid, bathed in fetid blood, lay chiefly in the vagina—a portion extended within the *os tincæ*. It was instantly and easily removed. The woman had bled to death, from a want of *skill* in the midwife.

Those accoucheurs greatly err, in regard to the delivery of the placenta, who say,—leave all to nature. This midwife had left all to nature, simply because she did not know how to deliver the placenta, the cord of which had been broken. Retained *placentæ*, when they fail to cause hemorrhages, sometimes bring on a fever like typhus. Retention, I am inclined to think, is occasioned by hour-glass contractions of the uterus oftner than is generally supposed. It is very surprising that Dr. Dewees never met, in his own practice, with a case of hour-glass contraction. I have met with a number. In two cases, the *placentæ* were retained so long that the ladies, as it were, forced me to deliver, after I tried ergot, &c. In the first case I was completely puzzled, not thinking at the moment of such a condition of the uterus—I explored its inner surface and found nothing—but on pulling the cord, I found it led through a firm strictured aperture, which I overcame according to the rules laid down in such cases; in the next case I found an ossific union between the placenta and the womb —a firm, cancillated network, crushing like the shelled bird's egg,—the whole being separated slowly, was brought away as usual in the hand. Never have I witnessed any pain, or ill effects, from introducing the hand and wrist into the uterus upon such occasions. Retained *placentæ* give rise to putrefactive odor of the most offensive kind: the sooner they can be got away the better for all parties.

Probably no town in the United States of equal population presents so much bad midwifery as New Orleans. Here, unlike other places, the evil of mal-practice falls not on the poor alone, but also on the rich, who often employ negresses that happen to be lucky. The late Judge Waggaman, formerly of the United States Senate, informed me, that some years since an old, drunken negress, who was a midwife on his plantation, being called to a young black woman in her first labor, took a sharp case knife, and performed the Cæsarean section, taking out a living child! The mother recovered soon, and had no inconvenience of a permanent kind, except a slight incontinence of urine. If this lucky negress had performed this operation for a rational end, obstetrical surgeons might have trembled for their laurels, since, according to Merryman and Blundell, it has been performed only twenty-six times in the British Isles, and has proved fatal to the mothers with but two exceptions.

Nearly all the white midwives of New Orleans are from foreign countries, and, with a very few exceptions, uneducated in their profession; and, as the law is becoming a dead letter, so far as medical practice is concerned, the number is augmenting rapidly. The law has done everything necessary to correct the evils of quackery: it requires in all branches of medicine, and in both sexes, education, examination, and license. The Faculty, or the Medical Board, or both, are to be blamed. It is hoped that they may yet be galvanized into life before all is lost.

"Midwives in France," says Dr. Stewart, "after two year's study at the School of Delivery, and submitting to two satisfactory examinations, receive a diploma to practise, always, however, under certain restrictions, one of which is, that in no case, and under no circumstances whatever, shall they resort to delivery with instruments without the attendance of a physician. They amount, in all France, to 450, and practise almost exclusively among the lower classes of the community." (Hospit. Paris, 1843.) This number, divided into the whole population, will give an average of one midwife to every 75,000 inhabitants; while New Orleans has, probably, one for every thousand,—nineteen in twenty of whom, it is supposed, could not give an account of the bones of the pelvis, or its diameters; of the womb, or its annexiæ; to say nothing of the general mechanism of parturition. In one respect the negresses are more safe than a vast majority of the German and Irish midwives—they will condescend to consult with doctors when difficulties arise, &c.

A few years ago, I was called to the mother of a considerable family, in labor with a monster more extraordinary in size and shape than perhaps any described by the late Geoffrey St. Hilaire—a monster in which it would be difficult to trace that unity of organization which this naturalist has shown to prevail even in the deviations of structure, designated as *lusus naturæ*—being three-legged, four-footed, double-spined, single-ribbed; in the trunk and viscera, single; in heads, necks, and arms, double; massive, unyielding, and probably weighing twenty-five to thirty pounds. The woman bathed in cold sweat; breathing quick; hiccupping; pulse and vital forces failing. A demented, or, at least, a stupid midwife was pulling by one leg, which she had found. Although there was no hope from the Cæsarean section, or from any source whatever the monster was carefully dissected as far as I could reach—along its posterior spine, corresponding to the maternal spine, to the shoulders of the monster; its anterior spine, corresponding to the maternal symphysis pubis, was removed about half-way up; one arm was obtained with difficulty, but the mass, which lay beyond the reach of instruments, was immovable, and the woman was dying, and soon expired. The Cæsarean section was performed. The four arms, upper part of the trunk, two necks with large heads—the foreheads directly opposite each other, formed a mass like an inverted cone, the apex of which was formed by the presenting parts, or legs,—a mass much of which was quite above the superior strait of the pelvis.—B. Dowler, M. D., *N. Y. Jour. of Medicine.*

